# The critical role of point defects in improving the specific capacitance of δ-MnO_2_ nanosheets

**DOI:** 10.1038/ncomms14559

**Published:** 2017-02-23

**Authors:** Peng Gao, Peter Metz, Trevyn Hey, Yuxuan Gong, Dawei Liu, Doreen D. Edwards, Jane Y. Howe, Rong Huang, Scott T. Misture

**Affiliations:** 1 Kazuo Inamori School of Engineering, Alfred University, Alfred, New York 14802, USA; 2 Hitachi High-Technologies Canada, Inc., 89 Galaxy Blvd, Suite 14, Toronto, Ontario, Canada M9W 6A4; 3 Cornell High Energy Synchrotron Source, Cornell University, Ithaca, New York 14853, USA

## Abstract

3D porous nanostructures built from 2D δ-MnO_2_ nanosheets are an environmentally friendly and industrially scalable class of supercapacitor electrode material. While both the electrochemistry and defects of this material have been studied, the role of defects in improving the energy storage density of these materials has not been addressed. In this work, δ-MnO_2_ nanosheet assemblies with 150 m^2^ g^−1^ specific surface area are prepared by exfoliation of crystalline K_*x*_MnO_2_ and subsequent reassembly. Equilibration at different pH introduces intentional Mn vacancies into the nanosheets, increasing pseudocapacitance to over 300 F g^−1^, reducing charge transfer resistance as low as 3 Ω, and providing a 50% improvement in cycling stability. X-ray absorption spectroscopy and high-energy X-ray scattering demonstrate a correlation between the defect content and the improved electrochemical performance. The results show that Mn vacancies provide ion intercalation sites which concurrently improve specific capacitance, charge transfer resistance and cycling stability.

## Abstract (web summary)

Two-dimensional solids are of interest for energy storage due to their large accessible surface area, enabling rapid charge/discharge. Here, the authors quantify the point defects in oxide nanosheets, demonstrating that intentional introduction of charged point defects improves the charge storage behaviour.

Manganese dioxide (MnO_2_) in its many forms has been the subject of much study for electrochemical capacitor applications^1^,^2^. In general, supercapacitors can be classified into two types: (i) electrical double-layer capacitors, which depend on charge separation at the electrode/electrolyte interface without Faradic process; and (ii) pseudocapacitors, which depend on Faradic redox reactions^3^,^4^,^5^. ‘Extrinsic' pseudocapacitance has recently emerged as a subclassification of materials that host ion intercalation but are engineered to short length scales to reduce diffusion distances such that the discharge behaviour becomes linear and no structural phase changes occur^6^,^7^.

The birnessite form of MnO_2_ (δ-MnO_2_), comprising stacked sheets of edge-shared MnO_6_ octahedra with interlayer alkali ions^8^,^9^,^10^, has been studied for some time and shows both double-layer and Faradaic charge storage^1^,^11^,^12^. Two-dimensional (2D) δ-MnO_2_ generally exhibits improved capacitance and rate behaviour compared to other polymorphs because the interlayer galleries provide high-speed pathways for diffusion of protons or alkali cations during the charge and discharge processes^13^,^14^,^15^. However, the low electrical conductivity of δ-MnO_2_ (10^−5^ to 10^−6^ S cm^−1^) has greatly limited its application^16^, prompting study of composite electrodes containing graphene^17^,^18^, carbon nanotubes^19^,^20^, carbon fibres^21^,^22^ and so on. In addition, nanostructuring has been employed to improve the surface area and capacitance, for example by growing nanoneedles^23^, nanoflowers^24^, nanoparticles^25^ and so on.

In recent years, intentional creation of cation vacancies has been explored to increase the charge storage capacitance of transition metal oxides, where cation vacancies provide additional cation intercalation sites^26^. Cation vacancy content may be controlled via aliovalent cation substitution^27^, anion substitution^28^, reducing or oxidizing heat treatments^29^, or by equilibrating the oxides in pH-controlled suspensions^30^. The first studies correlating cation vacancies and charge storage properties were published by Ruetschi in the mid-1980s for intergrowth γ-MnO_2_ phases^31^,^32^,^33^. Metal vacancy content can be quite large in some cases, for example, Wei *et al*.^28^ modified anatase TiO_2_ with monovalent F^−^ and OH^−^ anions to form up to 22 at% 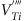 for additional Li storage. Similarly, Koo *et al*.^34^,^35^ transformed Fe_3_O_4_ (spinel) into hollow γ-Fe_2_O_3_ rods containing up to 44% vacant iron sites, and showed that Li and Na ion intercalation is possible without structural phase transformations.

Little work on the formation of cation vacancies in δ-MnO_2_ nanosheets has been reported to date, but extensive study of the important role of birnessite-like MnO_2_ in photosynthesis, ion sorption and other bio- and geochemical processes provides rich literature on its behaviour in aqueous environments^36^. Most recently, Manceau *et al*.^30^, building from many earlier works on Mn oxidation state in K-birnessites^10^,^37^, systematically investigated the effects of pH on cation vacancy content in phyllomanganate nanoparticles. Their approach exploited high-energy X-ray scattering to perform pair distribution function (PDF) analysis, truncation rod analysis and simulations using the Debye equation to show that lower pH causes migration of Mn from the MnO_2_ nanosheet to the sheet surface. Thus, the authors were able to quantify Mn vacancies in the nanosheets and Mn^3+^ cations displaced to the interlayers. Later work by Marafatto and coworkers^36^ employed sub-picosecond optical and X-ray absorption spectroscopy to track the mechanisms of Mn reduction under illumination to quantify the effects of different interlayer cations on MnO_2_ photo-reduction rates. Their results support the Mn redox reaction mechanism proposed by Manceau *et al*.^30^, including displacement of the Mn^3+^ cation to the interlayer gallery.

Additional recent work has been focused on determining mechanisms of charge storage. For example, *in situ* X-ray absorption studies have been used to show when the Faradaic reactions occur in MnO_2_ nanosheets^38^ and to track the average Mn oxidation state across wide voltage swings^39^,^40^,^41^. Similarly, *in situ* Raman spectroscopy has been applied to track changes in the vibrational bands and has highlighted cation size effects over the Li, Na and K series^42^.

In the present work, we exfoliate and reassemble δ-MnO_2_ nanosheets to form 3D macroporous pseudocapacitive electrodes with controlled concentration of Mn point defects and Mn^3+/4+^ ratios. Electrochemical and high-energy X-ray scattering measurements provide direct evidence that intentional Mn ion defects and Mn reduction synergistically improve supercapacitor performance. The three-dimensional (3D) assembly and defect control represent straightforward and industrially scalable approaches to improving specific capacitance.

## Results

### Phases and microstructure

**Figure 1:**
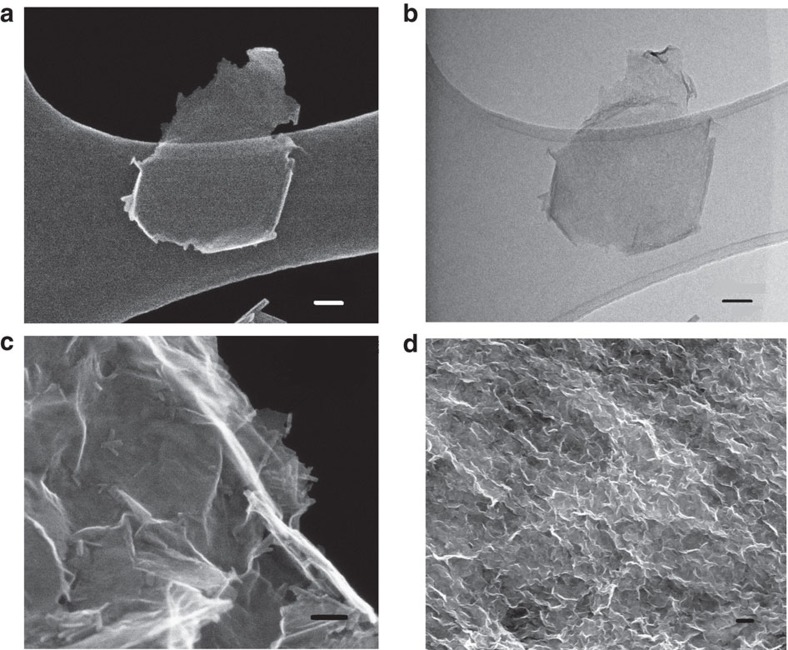
Electron microscopy of δ-MnO_2_ nanosheets. (**a**) SEM image and (**b**) bright-field TEM image of exfoliated MnO_2_ nanosheets, (**c**) high-magnification SEM image and (**d**) SEM image of reassembled MnO_2_ nanostructures treated in pH=2 solution for 24 h. Scale bar, 50 nm (**a**–**c**); 500 nm (**d**).

The morphologies of pristine K_*x*_MnO_2_, and its protonated form, H_*x*_MnO_2_, are shown in Supplementary Fig. 3. From Supplementary Fig. 3a,b it can be seen that the K_*x*_MnO_2_ and H_*x*_MnO_2_ particles are platy, with lateral dimensions in the range of one micron, and that proton exchange does not alter the grain morphology. High-magnification SEM and TEM images are shown in Fig. 1a–c for an individual nanosheet (Fig. 1a,b) and the reassembled MnO_2_ nanostructure (Fig. 1c). The images demonstrate that the sheets are generally flat, with some scrolling at the edges as has been noted in other nanosheet studies^43^,^44^. The atomic force microscopy (AFM) image in Supplementary Fig. 4 also displays nanosheet fragments with flat surfaces and thickness of five nanosheets, suggesting that some of the nanosheets exfoliate into bunches or restack to a small extent upon drying. The flocculated nanosheet samples exhibit 3D porous nanostructures (Fig. 1d; Supplementary Fig. 3c) and equilibration at different pH values have no influence on their morphologies. The observation of porous structures after reassembly highlights the usefulness of our ultrasonic-assisted exfoliation and flocculation procedure, where the exfoliation rate is greatly enhanced compared with other reported procedures^45^,^46^.

**Figure 2:**
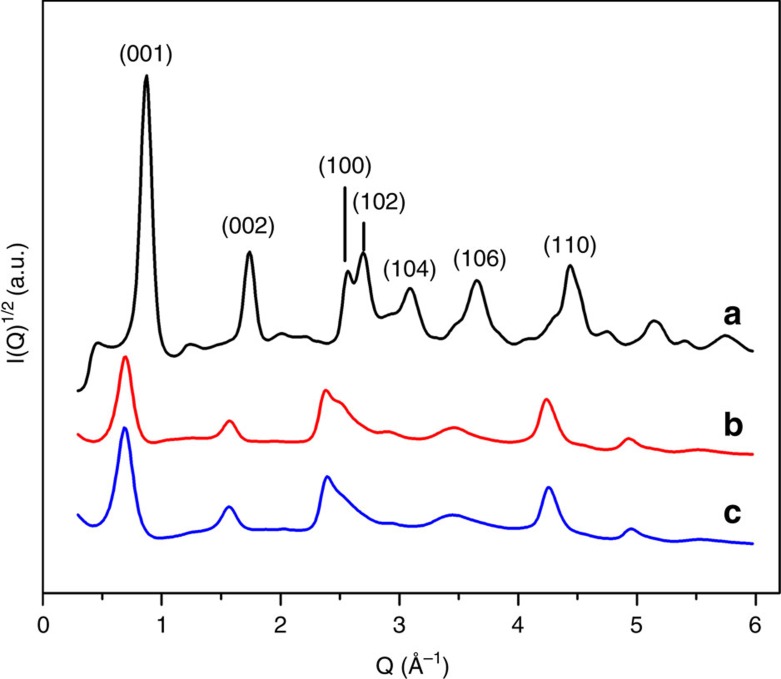
Powder diffraction patterns of protonated and reassembled δ-MnO_2_. XRD patterns of (**a**) protonated MnO_2_, (**b**) reassembled MnO_2_ treated in pH=4 solution for 24 h, (**c**) reassembled MnO_2_ treated in pH=2 solution for 24 h. Data collected on APS 11-ID-B.

Synchrotron diffraction data for the parent K_*x*_MnO_2_ is shown in Supplementary Fig. 5. Rietveld refinement reveals that the as-synthesized parent material is layered birnessite, primarily exhibiting the monoclinic polytype with less than 10 wt% of the rhombohedral polytype and no additional crystalline phases. The X-ray diffraction patterns (Fig. 2) for protonated and reassembled MnO_2_ demonstrate that phase purity was achieved during synthesis and that exfoliation and reassembly yields complex nanostructures as evidenced by peak broadening and asymmetry. After reassembly of the nanosheets, in-plane *hk0* reflections remain discernible, indicating that the crystalline nature of the 2D sheets is preserved. Further, the derived PDF is consistent both with literature^47^ and the calculated PDF of a single δ-MnO_2_ nanosheet (Supplementary Fig. 6), further confirming the δ-MnO_2_ nanosheet motif is maintained. The broadening of the 00*l* basal reflections shows that although some sheet-to-sheet restacking occurs, the stacks are on the order of only 3–4 nm (estimated using the Scherrer equation), which is consistent with the AFM results. Shifts of the basal reflections result from increased water content in the reassembled nanosheet floccules compared with the proton-exchanged form.

**Figure 3:**
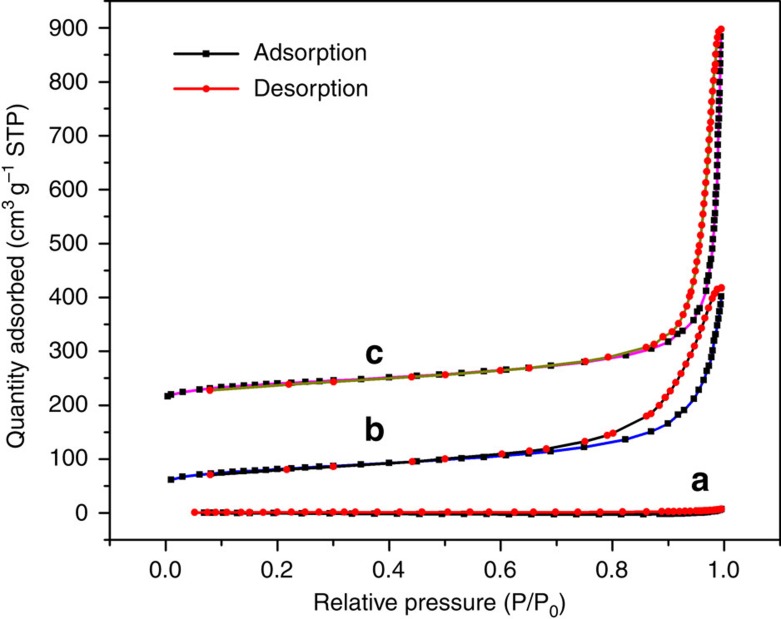
N_2_ adsorption isotherms of protonated and reassembled δ-MnO_2_. N_2_ adsorption–desorption isotherms of (**a**) protonated MnO_2_, (**b**) reassembled MnO_2_ treated in pH=2 solution for 24 h, (**c**) reassembled MnO_2_ treated in pH=4 solution for 24 h. Curves (**b**) and (**c**) are offset by 50 and 200 cm^3^ g^−1^ STP, respectively.

Nitrogen adsorption–desorption isotherms were used to quantify the specific surface area (SSA) of all specimens as shown in Fig. 3. While protonated MnO_2_ showed no evidence of mesopores, the reassembled MnO_2_ nanostructures show typical type IV isotherms (IUPAC classification) with distinct H3-type hysteresis loops, a result of open slit-like mesopores^48^. The Brunauer Emmet and Teller (BET) SSA were 120±0.4 m^2^ g^−1^ for the pH=2 sample and 144±1 m^2^ g^−1^ for the pH=4 sample, and only 4.5±0.1 m^2^ g^−1^ for H_*x*_MnO_2_, where the former values are roughly double the surface areas reported by Song *et al*.^49^ for reassembled oxide nanosheets. Altogether, the XRD, BET and microscopy show that the reassembled nanosheets have macro- and mesopores, with the mesoporosity arising due to loose agglomeration of randomly oriented sheet clusters. The extent of exfoliation and/or restacking, typical sheet thicknesses determined by AFM and average crystallinity in the sheet stacking direction as determined by XRD are similar or better for our MnO_2_ specimens than those reported recently for MnO_2_, TiO_2_, Co_3_O_4_, ZnO and WO_3_, for example^44^. Therefore, the typical structures in Fig. 1d are of the form of edge-to-face assembled nanosheet booklets, with ‘wall thicknesses' of up to 4 nm. The high SSA of the MnO_2_ nanosheet assemblies facilitates infiltration of the electrolyte into the porous electrode to enhance the specific capacitance^6^,^50^.

During soft chemical processing, the surface and interlayer tetrabutyl ammonium hydroxide (TBAOH) and water content may vary with processing conditions. The water content in protonated, pH=2 and pH=4 samples was obtained by thermogravimetric analysis (Supplementary Fig. 7). The presence of structural water enables rapid proton or alkali cation transport within the interlayer, which is beneficial for increasing the charge storage properties^6^. The slightly higher water content in the pH=4 versus pH=2 sample is a result of its slightly larger surface area. The presence of residual TBAOH in the reassembled samples was indicated by a small mass loss (∼3%, corresponding to 0.01 TBAOH per MnO_2_ formula unit and therefore about 9% surface coverage) in the temperature region of 200–400 °C (refs 51, 52). Infrared spectroscopy (Supplementary Fig. 8) and X-ray photoelectron spectroscopy (XPS) (Supplementary Fig. 9) were also used to detect remnant TBAOH in the nanosheet assemblies after processing. Infrared was used to show that the TBAOH is removed from the nanosheet assemblies after one charge–discharge cycle, as shown in Supplementary Fig. 10. Although it is not possible to completely remove the TBAOH molecules from the nanosheet assemblies by washing, its small surface coverage (∼9%) and easy extraction during the first electrochemical cycle together suggest that it has limited if any influence on the measured electrochemical performance.

### Redox and defects

**Figure 4:**
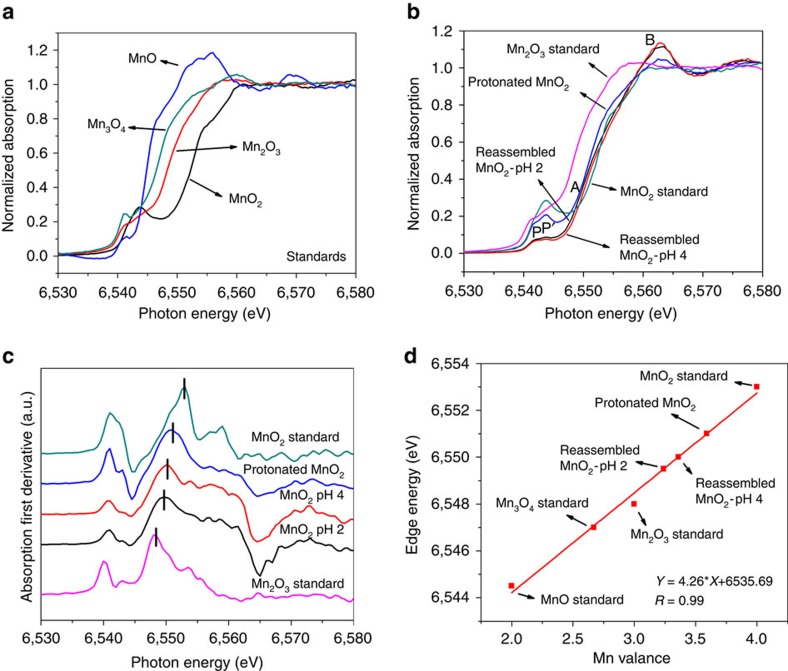
X-ray absorption spectra of MnO_*x*_ standards and experimental specimens. X-ray absorption measurements of as-prepared three samples and reference Mn oxide materials. (**a**) XANES spectra of reference materials MnO, Mn_3_O_4_, Mn_2_O_3_ and MnO_2_. (**b**) XANES spectra of protonated MnO_2_, pH=2 and 4 treated reassembled MnO_2_. The reference materials of Mn_2_O_3_ and MnO_2_ from (**a**) are also shown for of ease comparison. (**c**) First derivative curves corresponding to the samples shown in **b**. (**d**) Average oxidation state of Mn for the samples and standards derived from the K-edge energy.

The oxidation state of the Mn ions in all samples was investigated using Mn K-edge X-ray absorption near-edge spectroscopy (XANES) and XPS. The XPS analysis is described in Supplementary Information (Supplementary Fig. 11). Figure 4a shows the edge spectra of standard materials including MnO, Mn_3_O_4_, Mn_2_O_3_ and MnO_2_. The XANES spectra of the H_*x*_MnO_2_, pH=2 and 4 nanosheet assemblies, and reference materials Mn_2_O_3_ and MnO_2_ are shown in Fig. 4b. The line profiles are characterized by features that correspond to a pre-edge range with two weak broad peaks at 6,540–6,545 eV, a main-edge range that has one inflection point A, and the resonance peak range B^49^,^53^. The weak pre-edge peaks P and P′ correspond to the dipole-forbidden 1s→3d transition^54^. All three samples exhibit higher intensity of peak P′ than for peak P, but the peak P′ is less intense as compared with the β-MnO_2_ reference. This observation confirms the existence of mixed oxidation states of Mn^3+^/Mn^4+^ in the samples. Also, the higher intensity ratio of P′ to P for H_*x*_MnO_2_ compared with the pH=2 and 4 samples indicates that it has less Mn^3+^, because the relative intensity of the peaks P′/P is proportional to the average oxidation state^54^.

The main absorption range can be assigned to the dipole-allowed 1s→4p transition. The associated edge energy is usually taken as the energy of the peak in the first derivative, which corresponds to the inflection point of the main edge in the XANES spectra (Fig. 4c). Clearly, the main absorption edge (A) progressively shifts to lower energies with decreasing pH, implying lower pH progressively reduces Mn to the trivalent state. Also, the presence of the intense peak B for the nanosheet assemblies indicates that they are mainly comprised of edge-shared MnO_6_ octahedra^55^. This observation further confirms that the δ-MnO_2_ lattice is not dissolved by equilibration in HCl at pH as low as 1.

The average oxidation state (AOS) of Mn was determined by establishing a linear relationship between the K-edge energy and Mn oxidation state (Fig. 4d). The AOS of Mn is 3.59 for H_*x*_MnO_2_, 3.36 for the pH=4 MnO_2_ nanosheet assembly, and 3.24 for the pH=2 variant. These values are also listed in Table 1 for correlation with the PDF and electrochemical results discussed below. The dependence of Mn valence on pH generally follows that reported earlier by Manceau and coworkers^30^ for bulk Na-saturated δ-MnO_2_ (birnessite) powders, where the Mn AOS varied from 3.81 at pH=9 to 3.69 for pH=3. For the alkali-free MnO_2_ samples studied herein, we find that treating the exfoliated MnO_2_ assemblies at lower pH increases the Mn reduction to a greater extent than found for crystalline Na_*x*_MnO_2_ by Manceau and coworkers^30^. Furthermore, we find competing steric and thermodynamic effects by comparison of the proton-exchanged H_*x*_MnO_2_ to the exfoliated and reassembled variants. The AOS of H_*x*_MnO_2_ treated at pH<1 is 3.59, whereas the exfoliated samples treated at higher pH have AOS values of 3.36 (pH=4) and 3.24 (pH=2). We attribute the lesser extent of Mn reduction in crystalline H_*x*_MnO_2_ to steric hindrance by the interlayer galleries which are crowded with protons and water. This has the two-fold effect of hindering access of aqueous H_3_O^+^ to reducible Mn^4+^, and of preventing displacement of Mn^3+^ to the sheet surface. Indeed, earlier work by Gaillot *et al*.^10^ noted that during thermal reduction of 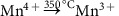 in crystalline K-birnessite, Mn vacancies were not formed despite the unfavourable in-sheet lattice strain due to Jahn–Teller distortions inherent to Mn^3+^. The interactions of neighbouring in-plane Mn^3+^ and Mn^4+^ sites therefore contributes both strain and electrostatic-driven components to the energetics of δ-MnO_2_ defect equilibration.

High-energy X-ray scattering and pair distribution function analysis were undertaken to further characterize the defects formed in soft chemically reduced MnO_2_ nanosheets, with the specific goal of correlating the quantity of Mn point defects with Mn reduction and electrochemical performance. PDF analysis probes not only the local atomic bonding motifs but also intermediate and long-range order, thus making it an appropriate tool for investigating the structures of poorly-crystalline materials and nanoparticles that yield diffraction patterns with large amounts of diffuse scattering^47^.

**Figure 5:**
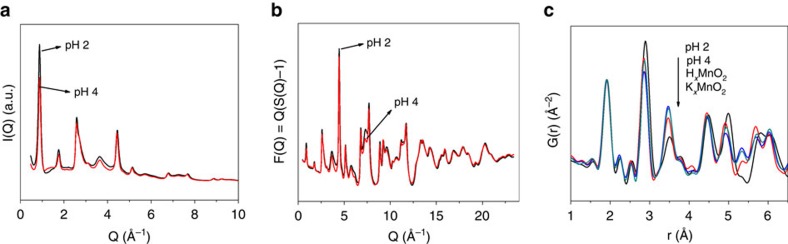
High-energy X-ray scattering and experimental PDF data. (**a**) I(Q) and (**b**) F(Q) for the pH=2 and pH=4 treated MnO_2_ nanosheets assemblies, and (**c**) G(r) for the pH-treated samples, protonated MnO_2_ (H_*x*_MnO_2_), and parent phases (K_*x*_MnO_2_). The basal reflections evident in I(Q<2 Å^−1^) indicate a measure of restacking. The variation in intensity as a function of pH indicates a variation in the interlayer structure attributable to either surface water or Mn. The inverse trends in the Mn–Mn peak (2.89 Å) and Mn–Mn^IL^ peak (3.45 Å) as a function of pH support the Mn^3+^ displacement model recommended by Manceau *et al*.^30^

**Figure 6:**
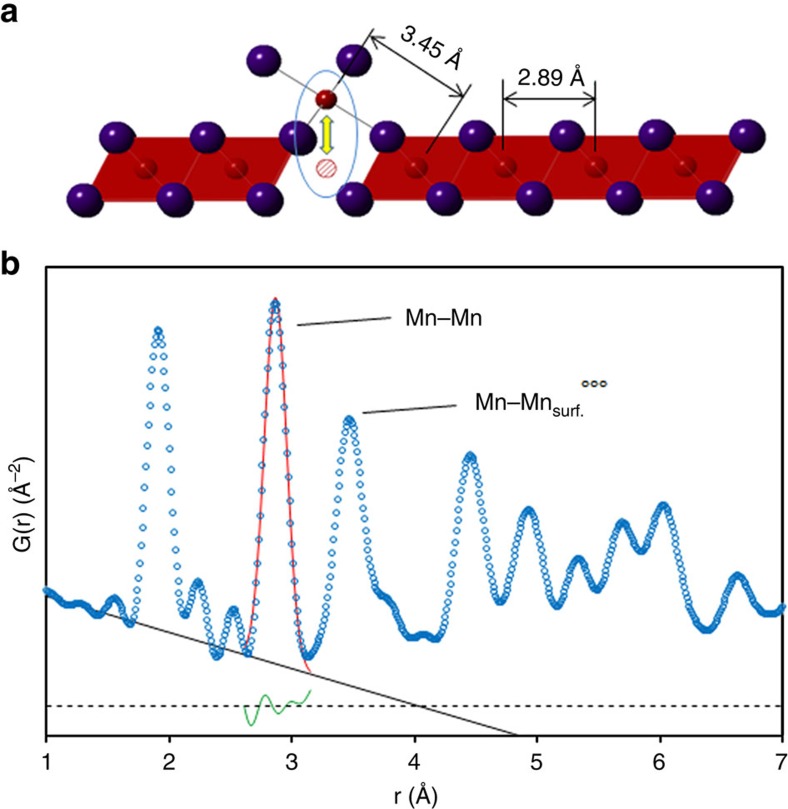
Empirical analysis of δ-MnO_2_ PDF. Using the resolved in-plane Mn-Mn correlation peak (**a**) we can empirically estimate the Mn surface Frenkel (circled) concentration using a Gaussian peak and linear baseline (**b**). The PDF amplitude is normalized to the Mn–O correlation peak; therefore the ratio of nanosheet Mn-Mn to K_x_MnO_2_ Mn-Mn peak areas represents the fractional Mn-occupancy of the nanosheet assembly. δ-MnO_2_ equilibrated at pH=2 is shown here.

The observed scattered intensity, the reduced structure function F(Q) and the associated PDF for the reassembled nanosheets are shown in Fig. 5. We focus our attention on two PDF correlation peaks: the in-plane Mn–Mn peak at 2.89 Å and the Mn–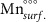 distance at 3.45 Å (Fig. 6a). Two notable advantages of the PDF method are that these two Mn correlations are unique in the alkali-free δ-MnO_2_ structure (Fig. 6b) and that the integrated area of a PDF peak represents the number density of that specific correlation type^56^. We leverage these facts to obtain useful estimates of Mn vacancy concentrations in lieu of developing complete structure models, which are notably difficult for these material systems^57^,^58^.

To compare the PDF with respect to different samples, we have normalized our data to the amplitude of the 1.9 Å Mn–O peak. This choice is based on the implicit assumption that all Mn ions are coordinated in a six-fold environment. We further assume that the intrinsic 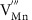 population in the parent phase K_x_MnO_2_ is on the order of parts per million, which will be negligible compared to the per cent-level vacancies in the MnO_2_ nanostructures. Consequently, the relative in-plane Mn concentration can be calculated according to the following equation:


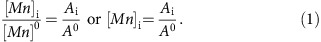


Where the superscript ^0^ denotes quantities in K_*x*_MnO_2_, the subscript _i_ denotes quantities for a derivative sample, and [*Mn*]^0^ is taken as 1. The absolute accuracy of the values obtained using equation (1) will be influenced by the correlations between interlayer water or hydroxyl pairs, which perturb the low-r PDF region, leading to overestimation of the 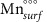 content^47^. While there is notable difficulty in absolute quantification of Mn defects in these materials, this method should provide reasonable relative quantification in the derived nanosheet assemblies. Although estimation of error is not possible with this empirical approach, a per cent level of uncertainty is expected for the calculated Mn concentrations.

As shown in Fig. 6a, the 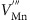 concentration, which is equivalent to the 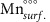 concentration, is then is 1−[*Mn*]_i_ in fractional units. Here we define this type of defect as a ‘surface Frenkel' defect, where displacement of the in-plane Mn to the nanosheet surface is reminiscent of the Frenkel defect vacancy-interstitial pair. The results of our analysis are summarized in Table 2. Consistent with the model of Manceau *et al*.^30^, we observe an increase in 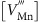 with decreasing pH, as well as the appearance of a PDF peak at a distance not found in the δ-MnO_2_ structure, which corresponds to 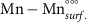. The increase in concentration of surface Frenkel defects by ∼30% between the pH=4 and pH=2 samples supports the hypothesis that increased proton sorption at the MnO_2_ surface in more acidic electrolytes expels more in-plane Mn leading to the formation of more Mn vacancies.

As noted for the Mn valence, the PDF analysis likewise shows an apparent contradiction in the surface Frenkel defect content of the crystalline H_*x*_MnO_2_ when compared to the defect content of the pH=2 and 4 samples. As shown in Table 1, while the protonated form H_*x*_MnO_2_ is equilibrated at pH<1, its defect concentration (18.3%) is smaller than that in the pH=2 sample (26.5%) and is even smaller than that of the pH=4 sample (19.9%). As noted earlier, previous work shows that Mn^3+^, with its Jahn–Teller distortion, may be accommodated in MnO_2_ sheets in crystalline birnessites^59^. Table 1 shows that ∼2/3, 1/2 and 1/4 of the Mn ions remaining in the nanosheets are reduced to Mn^3+^ for the pH=2, 4 and H_*x*_MnO_2_ samples, respectively. The PDF and XANES analyses are therefore complementary in demonstrating that surface Frenkel defects and Mn reduction are more favourable in high-surface area nanosheet assemblies, with steric limitations reducing the extent of the reactions in well-crystalline birnessites.

### Electrochemical measurements

**Figure 7:**
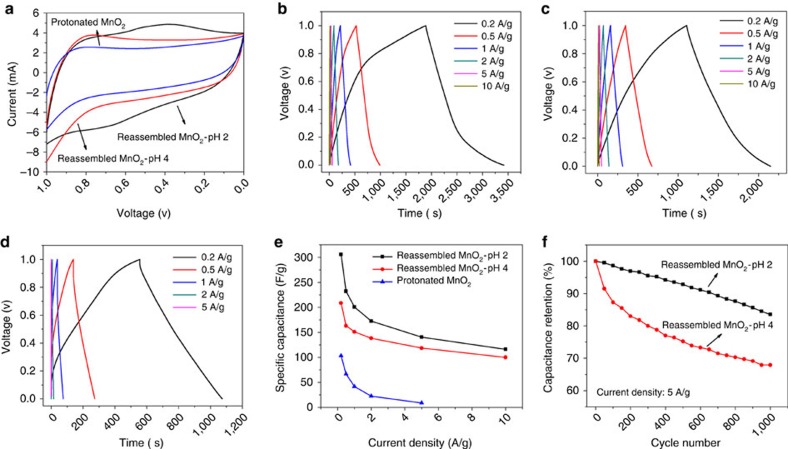
Electrochemical performance of reassembled δ-MnO_2_ electrodes. Electrochemical testing results: (**a**) CV curves for the samples at 50 mV s^−1^ scan rate; (**b**), (**c**) and (**d**) galvanostatic charge–discharge curves of the pH=2, 4 and H_*x*_MnO_2_ samples; (**e**) comparison of specific capacitance for the samples as a function of current density; and (**f**) cycle stability at constant current density of 5 A g^−1^ between 0 and 1 V.

As shown in Fig. 7a, the cyclic voltammetry (CV) curves for all three samples exhibit largely rectangular shapes in the potential window from 0 to 1 V, which indicates capacitive behaviour. The absence of clear redox peaks for protonated and pH=4 samples implies that the electrodes are charged and discharged at a pseudo-constant rate over the whole voltammetric cycle. However, the presence of broad redox peaks for the pH=2 sample indicates that ion intercalation is an active and detectable charge storage mechanism for this specimen. Indeed, even broader redox peaks are noted for the pH=3 sample (Supplementary Fig. 12a), while no redox peaks are found for the pH=4 and pH=9 (Supplementary Fig. 12b) samples. The marked increase in Mn surface Frenkel defects with decreasing pH appears to enhance the intercalation reaction. This is also reflected in Fig. 7b–d, which shows the galvanostatic charge–discharge curves for the samples between 0 and 1 V under different current densities. The potential does not linearly change with time, a behaviour typical for pseudocapacitive materials that involve redox reactions.

The specific capacitance of the electrode measured by the galvanostatic discharge method can be calculated as:


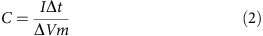


where *C* (F g^−1^) is the specific capacitance, *I* (A) is the constant discharge current, Δ*t* (s) is the discharge time, Δ*V* (V) is the potential window, and *m* (g) is the mass of the active material in the electrode. The specific capacitances of the three samples obtained at different current densities are summarized in Fig. 7e. At 0.2 A g^−1^, the specific capacitance is 306 F g^−1^ for the pH=2 sample, 209 F g^−1^ for the pH=4 sample and 103 F g^−1^ for crystalline H_*x*_MnO_2_. With increasing current density, the specific capacitance decreases gradually for all the three samples.

The defect chemistry also has an impact on cycling stability, demonstrated using the pH=2 and 4 nanosheet assemblies, which is shown in Fig. 7f. After 1,000 charge–discharge cycles using a very high-current density of 5 A g^−1^ (2.5 mA cm^−2^), the pH=2 sample shows good stability, retaining 83% of its initial specific capacitance. In contrast, the pH=4 sample retains only 68% of its initial capacitance. Our ongoing work involves tracking the reversibility of the electrochemical strains induced during cycling, and although we have not yet uncovered the origins of the cycling fade, our results are promising when compared with earlier work, for example for MnO_2_ nanostructures which retain ∼92% of the initial capacity after 1,000 cycles when using a current density five times smaller^49^.

**Figure 8:**
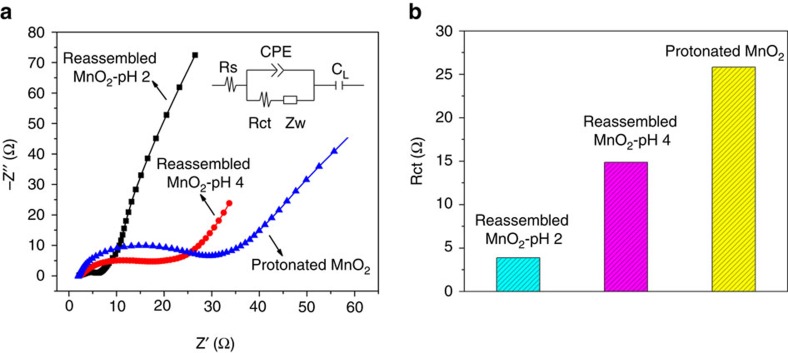
Electrochemical impedance spectroscopy of reassembled δ-MnO_2_ electrodes. (**a**) Nyquist plots of the samples in the frequency range of 0.1 Hz–100 kHz at an open circuit potential of 5 mV (inset shows the electrical equivalent circuit used for fitting the impedance spectra); and (**b**) comparison of the charge transfer resistance among the three samples (values obtained from the fitted data).

Electrochemical impedance spectroscopy (EIS) was employed to measure the charge transfer resistance of each electrode with the results shown in Fig. 8a. All plots exhibit a straight line in the low-frequency region and a single semicircle in the high-frequency region, indicating a diffusion-limited step in the low frequency region and a charge transfer limited step in the high-frequency region^60^. The Nyquist plots were further modelled and interpreted by using an appropriate electrical equivalent circuit, which is shown in the inset in Fig. 8a. *R*_*s*_ is a combined resistance including ionic resistance of the electrolyte, intrinsic resistance of the substrate and contact resistance at the active material/current collector interface. *R*_*ct*_ is the charge transfer resistance caused by the Faradaic reaction. *Z*_*w*_ is the Warburg resistance which is related to ion diffusion in the electrolyte, *CPE* is a constant phase element and *C*_*L*_ is the limit capacitance^61^,^62^,^63^. The calculated charge transfer resistances (*R*_*ct*_) extracted from the high-frequency range were 23, 15 and 3 Ω for the H_*x*_MnO_2_, pH=4 and pH=2 samples, respectively, as shown in Fig. 8b.

Table 1 summarizes the characteristics of the electrodes and their properties, showing several clear trends. First, the XPS and XANES studies, combined with quantification of surface Frenkel defects, demonstrate that the Mn^3+/4+^ ratio in the nanosheets trends with pH treatment. More specifically, Mn^4+^→Mn^3+^ reduction does not only result in formation of surface Frenkel defects but some of the Mn within the sheets is reduced to the trivalent state. As calculated based on the data in Table 1, ∼2/3, 1/2 and 1/4 of the Mn ions remaining in the nanosheets are reduced to Mn^3+^ for the pH=2, 4 and H_*x*_MnO_2_ samples, respectively.

The specific capacitance for the reassembled nanosheets correlates with the surface Frenkel defect population and Mn^3+^ content, as well as charge transfer resistance. For example, the surface areas of the pH=2 and 4 samples are similar, but the sample treated at pH=2 has 50% higher capacitance, 47% more Na^+^ ion intercalation, × 5 smaller charge transfer resistance, with 33% more surface Frenkel defects, highlighting the importance of the cation defects on Na^+^ ion intercalation.

The surface Frenkel defect provides two likely intercalation sites: the Mn ion vacancy, which is accessible from one side of the nanosheet; and the undercoordinated MnO_6_ surface octahedron on the opposite side of the nanosheet (Fig. 6a). In parallel, the defect reaction provides a large concentration of Mn^3+^ cations, which can presumably participate in polaron hopping conduction, thus improving electrical conductivity and charge transfer efficiency^64^,^65^. The specific mechanism of Na^+^ ion intercalation into the nanosheets is a topic of current study in our group, but we speculate that there exists some synergistic effect of the defect content and Mn redox that together define the charge transfer resistance and specific capacitance. The electrochemical cycling behaviours suggest that is likely that steric effects such as local distortions in the nanosheets around vacancies are active in relieving electrochemical strains that occur during cycling, and thus define to a large extent the specific capacitance.

## Discussion

Electrostatic assembly of δ-MnO_2_ nanosheets in suspension under carefully controlled experimental conditions yields self-assembled 3D porous nanostructures with surface areas of ∼150 m^2^ g^−1^. Equilibrating the reassembled nanosheets in varied pH controls the extent of Mn^4+^→Mn^3+^ reduction, as well as creating charged defect pairs we term ‘surface Frenkel defects' comprising a Mn vacancy within the sheet and a six-fold coordinated Mn^3+^ site on the surface of the nanosheet. The Mn surface Frenkel defect content reaches 26.5% for the nanosheet assemblies equilibrated at pH=2 and 19.9% for the pH=4 sample. The XPS and XANES data indicate an increase of the Mn^3+^/Mn^4+^ ratio with decreasing pH and the electrochemical results show direct correlation of Mn cation defects with specific capacitance. The specific capacitance increased from about 200 F g^−1^ (pH=4) to over 300 F g^−1^ (pH=2) by intentional introduction of ∼30% surface Frenkel defects, while at the same time the charge transfer resistance decreased from ∼15 Ω to ∼3 Ω. Therefore, it is now clear that Mn surface Frenkel defects in δ-MnO_2_ nanosheets increase Na^+^ ion intercalation by providing new, low energy intercalation sites.

## Methods

### Chemicals and reagents

MnCO_3_, K_2_CO_3_, Na_2_SO_4_, acetylene black, poly (vinylidene fluoride) (PVDF) and nickel foil were purchased from Alfa Aesar. 6N hydrochloric acid (HCl) solution and sodium hydroxide (NaOH) were obtained from Fisher Scientific. The tetrabutylammonium hydroxide solution (TBAOH, 40 wt% in H_2_O) and N-methyl-2-pyrrolidone (NMP) were purchased from Sigma-Aldrich. All reagents were used as received without further purification.

### Fabrication of δ-MnO_2_ nanostructures

Powder synthesis included mixing MnCO_3_ and K_2_CO_3_ powders in a molar ratio of 40 : 9, by milling in isopropyl alcohol for 10 min using a McCrone Micronizing Mill (McCrone Group, USA) with alumina media. The resulting suspension was dried on a hot plate for 30 min at 60 °C and then calcined in alumina crucible at 800 °C for 24 h in air. 0.5 g of the resulting layered K_*x*_MnO_2_ was proton ion-exchanged in HCl solution (1 mol l^−1^, 45 ml) in an ultrasonic bath at room temperature for 4 h. The ion exchange process was repeated two additional times, followed by washing with DI water and air drying. The absence of K ions was confirmed by energy dispersive spectroscopy and XPS survey scans shown in Supplementary Figs 1 and 2. In addition, the XPS survey scans showed a complete absence of any signal from Al ions, giving good confidence that no contamination by the Al_2_O_3_ milling media was encountered.

To obtain exfoliated MnO_2_ nanosheets, 0.35 g H_*x*_MnO_2_ was equilibrated with 32.5 ml aqueous TBAOH solution (30 ml H_2_O+2.5 ml 40 wt% TBAOH) for 4 h in an ultrasonic bath at room temperature. The resulting suspension was centrifuged at 10,000 r.p.m. for 10 min. to separate the remaining bulk H_*x*_MnO_2_ from the nanosheet suspension. Self-assembly of the nanosheets in the colloidal suspension was achieved by adding 6N HCl solution to the suspension at a constant rate of 1 ml min^−1^, while stirring to reach pH=2, resulting in flocculation to form high-surface area 3D porous structures.

Mn defects and redox were controlled by equilibrating the assembled nanosheets at target pH values by increasing the pH from 2 upwards using 1 mol l^−1^ NaOH additions and stirring for 24 h. Dry nanosheet assemblies were finally obtained by washing, centrifugation, rinsing in 2-propanol and drying overnight at room temperature. Thus, 3D porous MnO_2_ nanostructures assembled from ultrathin 2D δ-MnO_2_ nanosheets with controlled defect content were obtained. For comparison with the 3D assembled nanosheets, protonated bulk H_*x*_MnO_2_ without exfoliation was also electrochemically tested.

### Characterization of the samples

Microstructures were studied using scanning electron microscopy (SEM, FEI Quanta 200) at 20 kV. Transmission electron microscopy and high-resolution SEM were carried out using a Hitachi HF-3300 TEM/STEM. The STEM unit has a secondary-electron detector, which allows simultaneous high-resolution SEM and TEM imaging at 300 kV. The thickness and crystallite dimension of the exfoliated MnO_2_ nanosheets were probed by multimode atomic force microscope (AFM, Bruker Dimension Icon) in tapping mode using antimony doped silicon tips. The exfoliated nanosheets were electrostatically attached to a clean silicon wafer by drying the nanosheet suspension, and imaged in air. Thermogravimetric analysis (TGA) was performed using a TA Instruments SQT-Q600 DTA/TGA under flowing air with a heating rate of 10 °C min^−1^. The presence of any functional groups from organic compounds was probed with infrared spectroscopy (Nicolet 6700 FT-IR Spectrometer, USA). The specific surface area and porosity were examined by nitrogen adsorption and desorption isotherms collected at 77 K using a Micromeritics TriStar II 3020 system. The local chemical environment of the samples was characterized using a PHI Quantera X-ray photoelectron spectrometer equipped with Al Kα radiation. Mn K-edge X-ray absorption near-edge spectroscopy measurements were carried out at the bending magnet beamline F3 at the Cornell High Energy Synchrotron Source (CHESS). Data were collected at room temperature in fluorescence mode using a Hitachi vortex 4-element silicon drift detector. All spectra were calibrated using the spectrum of Mn metal foil, and the software package ATHENA was used for analysis^66^. The Mn vacancy content in δ-MnO_2_ samples was determined using high-energy X-ray scattering and PDF analysis, with data collected at the Advanced Photon Source on beam-line 11-ID-B using the rapid-acquisition PDF geometry^67^. Data sets were collected using standard 1 mm Kapton capillaries in Debye-Scherrer geometry, Si<311> monochromated primary beam at 58.66 keV, and a silicon flat plate area detector. Scattering data for PDF extraction were collected over a Q-range of 0.4–24.5 Å^−1^, and the powder diffraction data for all samples was collected over a Q-range of 0.2–9.0 Å^−1^. 2D X-ray diffraction data were integrated to 1D using FIT2D^68^, after appropriately calibrating detector deviations from orthogonality and masking invalid pixels. CeO_2_ was used as the calibration standard for detector geometry. Meanwhile, CeO_2_ and Ni were used to evaluate the instrument response function. The PDF data was reduced using PDFgetX2 (ref. 69), which includes the appropriate corrections for inelastic scattering and energy-dependent detector response, in addition to experimental background and absorption corrections, amongst others.

### Electrochemical measurements

The working electrode was prepared by mixing 80 wt% active material, 15 wt% acetylene black and 5 wt% PVDF in NMP solution. After stirring for 6 h, the homogeneous slurry was spread onto a Ni foil substrate with an area of 1 cm^2^, and then heated at 100 °C for 2 h to evaporate the solvent and obtain the electrode. The loading of the active material on the working electrode was in the range of 0.5–0.6 mg cm^−2^. The capacitive performance was measured using a CHI 650E electrochemical analyser (CHI, USA) with a conventional three-electrode cell. Ag/AgCl and platinum wire were used as the reference and auxiliary electrodes, respectively, with 1 M Na_2_SO_4_ aqueous electrolyte. Cyclic voltammetry scans were carried out from 0 to 1 V at a scan rate of 50 mV s^−1^. Galvanostatic charge–discharge was measured at different constant current densities from 0.2 to 10 A g^−1^. EIS was performed in the frequency range of 0.1 Hz–100 kHz at an open circuit potential of 5 mV. EIS data was fitted to an electrical equivalent circuit model using ZsimpWin (Version 3.21, EChem Software) software.

### Data availability

The data that support the findings of this study are available from the authors on request.

## Additional information

**How to cite this article:** Gao, P. *et al*. The critical role of point defects in improving the specific capacitance of δ-MnO_2_ nanosheets. *Nat. Commun.* **8,** 14559 doi: 10.1038/ncomms14559 (2017).

**Publisher's note:** Springer Nature remains neutral with regard to jurisdictional claims in published maps and institutional affiliations.

## Supplementary Material

Supplementary Information Supplementary Figures, Supplementary Table and Supplementary References

Peer Review File

## Figures and Tables

**Table 1:** Summary findings of defects and electrochemistry in δ-MnO_2_ electrodes.

**Samples**	**AOS**	**[Mn**^**3+**^**] (%)**	**[Mn**^**4+**^**] (%)**	**[*****V***_**Mn**_**] (%)**	**S.S.A.** **(m**^**2**^** g**^−**1**^**)**	***C***_***p***_ **(F g**^−**1**^**)**	***R***_***ct***_ **(Ω)**	**Na : Mn (fract.)**
Reassembled MnO_2_ - pH 2	3.24	76	24	26.5	120	306	3	0.28
Reassembled MnO_2_ - pH 4	3.36	64	36	19.9	144	209	15	0.19
Protonated MnO_2_	3.59	41	59	18.3	4.5	103	23	0.09

Comparison of defect structures and electrochemical supercapacitor properties of bulk protonated H_x_MnO_2_ and MnO_2_ nanosheet assemblies.

**Table 2:** Estimation of surface Frenkel defect concentration.

**Data set**	**Centre (Å)**	**Height (arb.)**	**Area (arb.)**	**FWHM (Å)**	**Mn-vac. (%)**
Pristine MnO_2_	2.897	15.988	3.461	0.203	N/A
Protonated MnO_2_	2.858	13.896	2.826	0.191	18.3
Reassembled MnO_2_–pH 4	2.876	13.810	2.772	0.189	19.9
Reassembled MnO_2_–pH 2	2.867	12.335	2.544	0.194	26.5

Gaussian parameters for the Mn–Mn in-sheet PDF peak corresponding to different samples extracted using Fityk^70^. Peak area is normalized to the Mn vacancy content of the parent phase K_*x*_MnO_2_ with the assumption that the intrinsic 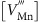 population is on the order of parts per million in the high-temperature crystalline phase.

## References

[b1] Ghodbane O., Ataherian F., Wu N.-L. & Favier F. *In situ* crystallographic investigations of charge storage mechanisms in MnO_2_ -based electrochemical capacitors. J. Power Sources 206, 454–462 (2012).

[b2] Wei W., Cui X., Chen W. & Ivey D. G. Phase-controlled synthesis of MnO_2_ nanocrystals by anodic electrodeposition: implications for high-rate capability electrochemical supercapacitors. J. Phys. Chem. C 112, 15075–15083 (2008).

[b3] Chen S., Zhu J., Wu X., Han Q. & Wang X. Graphene oxide-MnO_2_ nanocomposites for supercapacitors. ACS Nano 4, 2822–2830 (2010). 20384318 10.1021/nn901311t

[b4] Meng C., Liu C., Chen L., Hu C. & Fan S. Highly flexible and all-solid-state paperlike polymer supercapacitors. Nano Lett. 10, 4025–4031 (2010). 20831255 10.1021/nl1019672

[b5] Oh S. & Kim K. Synthesis of a new mesoporous carbon and its application to electrochemical double-layer capacitors. Chem. Commun. 1999, 2177–2178 (1999).

[b6] Augustyn V., Simon P. & Dunn B. Pseudocapacitive oxide materials for high-rate electrochemical energy storage. Energy Environ. Sci. 7, 1597–1614 (2014).

[b7] Augustyn V. . High-rate electrochemical energy storage through Li^+^ intercalation pseudocapacitance. Nat. Mater. 12, 518–522 (2013). 23584143 10.1038/nmat3601

[b8] Zhu H. . Birnessite-type MnO_2_ nanowalls and their magnetic properties. J. Phys. Chem. C 112, 17089–17094 (2008).

[b9] Ma R., Bando Y., Zhang L. & Sasaki T. Layered MnO_2_ nanobelts: hydrothermal synthesis and electrochemical measurements. Adv. Mater. 16, 918–922 (2004).

[b10] Gaillot A.-C., Lanson B. & Drits V. A. Structure of birnessite obtained from decomposition of permanganate under soft hydrothermal conditions. 1. Chemical and structural evolution as a function of temperature. Chem. Mater. 17, 2959–2975 (2005).

[b11] Pan H.-A. . Investigating mechanisms underlying elevated-temperature-induced capacity fading of aqueous MnO_2_ polymorph supercapacitors: cryptomelane and birnessite. J. Electrochem. Soc. 162, A5106–A5114 (2015).

[b12] Brousse T., Bélanger D. & Long J. W. To be or not to be pseudocapacitive? J. Electrochem. Soc. 162, A5185–A5189 (2015).

[b13] Vargas O., Caballero A., Hernán L. & Morales J. Improved capacitive properties of layered manganese dioxide grown as nanowires. J. Power Sources 196, 3350–3354 (2011).

[b14] Radhiyah A. . Doubling of electrochemical parameters via the pre-intercalation of Na^+^ in layered MnO_2_ nanoflakes compared to α-MnO_2_ nanorods. RSC Adv. 5, 9667–9673 (2015).

[b15] Komaba S., Ogata A. & Tsuchikawa T. Enhanced supercapacitive behaviors of birnessite. Electrochem. Commun. 10, 1435–1437 (2008).

[b16] Zhang X., Yang W. & Ma Y. Synthesis of polypyrrole-intercalated layered manganese oxide nanocomposite by a delamination/reassembling method and its electrochemical capacitance performance. Electrochem. Solid-State Lett. 12, A95–A98 (2009).

[b17] Peng L. . Ultrathin two-dimensional MnO_2_/graphene hybrid nanostructures for high-performance, flexible planar supercapacitors. Nano Lett. 13, 2151–2157 (2013). 23590256 10.1021/nl400600x

[b18] Zhu J. & He J. Facile synthesis of graphene-wrapped honeycomb MnO_2_ nanospheres and their application in supercapacitors. ACS Appl. Mater. Interfaces 4, 1770–1776 (2012). 22329919 10.1021/am3000165

[b19] Hu L. . Symmetrical MnO_2_–carbon nanotube–textile nanostructures for wearable pseudocapacitors with high mass loading. ACS Nano 5, 8904–8913 (2011). 21923135 10.1021/nn203085j

[b20] Lee S. W., Kim J., Chen S., Hammond P. T. & Shao-Horn Y. Carbon nanotube/manganese oxide ultrathin film electrodes for electrochemical capacitors. ACS Nano 4, 3889–3896 (2010). 20552996 10.1021/nn100681d

[b21] Luo Y. . Self-assembly of well-ordered whisker-like manganese oxide arrays on carbon fiber paper and its application as electrode material for supercapacitors. J. Mater. Chem. 22, 8634–8640 (2012).

[b22] Chen Y.-C. . Highly flexible supercapacitors with manganese oxide nanosheet/carbon cloth electrode. Electrochim. Acta 56, 7124–7130 (2011).

[b23] Kong D. . Three-dimensional Co_3_O_4_@MnO_2_ hierarchical nanoneedle arrays: morphology control and electrochemical energy storage. Adv. Funct. Mater. 24, 3815–3826 (2014).

[b24] Zhao S. . Controlled synthesis of hierarchical birnessite-type MnO_2_ nanoflowers for supercapacitor applications. Appl. Surf. Sci. 356, 259–265 (2015).

[b25] Jiang R., Huang T., Liu J., Zhuang J. & Yu A. A novel method to prepare nanostructured manganese dioxide and its electrochemical properties as a supercapacitor electrode. Electrochim. Acta 54, 3047–3052 (2009).

[b26] Rolison D. R. & Nazar L. F. Electrochemical energy storage to power the 21st century. MRS Bull. 36, 486–493 (2011).

[b27] Hahn B. P. . Electrochemical Li-ion storage in defect spinel iron oxides: the critical role of cation vacancies. Energy Environ. Sci. 4, 1495–1502 (2011).

[b28] Li W. . High substitution rate in TiO_2_ anatase nanoparticles with cationic vacancies for fast lithium storage. Chem. Mater. 27, 5014–5019 (2015).

[b29] Swider-Lyons K. E., Love C. T. & Rolison D. R. Improved lithium capacity of defective V_2_O_5_ materials. Solid State Ionics 152, 99–104 (2002).

[b30] Manceau A. . Short-range and long-range order of phyllomanganate nanoparticles determined using high-energy X-ray scattering. J. Appl. Crystallogr. 46, 193–209 (2013).

[b31] Ruetschi P. Cation-vacancy model for MnO_2_. J. Electrochem. Soc. 131, 2737–2744 (1984).

[b32] Ruetschi P. Influence of cation vacancies on the electrode potential of MnO_2_. J. Electrochem. Soc. 135, 2657–2663 (1988).

[b33] Ruetschi P. & Giovanoli R. Cation vacancies in MnO_2_ and their influence on electrochemical reactivity. J. Electrochem. Soc. 135, 2663–2669 (1988).

[b34] Koo B. . Intercalation of sodium ions into hollow iron oxide nanoparticles. Chem. Mater. 25, 245–252 (2013).

[b35] Koo B. . Hollow iron oxide nanoparticles for application in lithium ion batteries. Nano Lett. 12, 2429–2435 (2012). 22468698 10.1021/nl3004286

[b36] Marafatto F. F. . Rate and mechanism of the photoreduction of birnessite (MnO_2_) nanosheets. Proc Natl. Acad. Sci. USA 112, 4600–4605 (2015). 25825757 10.1073/pnas.1421018112 PMC4403223

[b37] Gaillot A. C., Drits V. A., Manceau A. & Lanson B. Structure of the synthetic K-rich phyllomanganate birnessite obtained by high-temperature decomposition of KMnO_4_. Substructures of K-rich birnessite from 1,000 °C experiment. Micropor. Mesopor. Mater. 98, 267–282 (2007).

[b38] Yeager M. . Highly efficient K_0.15_MnO_2_ birnessite nanosheets for stable pseudocapacitive cathodes. J. Phys. Chem. C 116, 20173–20181 (2012).

[b39] Nam K.-W., Kim M. G. & Kim K.-B. *In situ* Mn K-edge X-ray absorption spectroscopy studies of electrodeposited manganese oxide films for electrochemical capacitors. J. Phys. Chem. C 111, 749–758 (2007).

[b40] Ma S.-B. . Electrochemical properties of manganese oxide coated onto carbon nanotubes for energy-storage applications. J. Power Sources 178, 483–489 (2008).

[b41] Weng Y. T. . Spatially confined MnO_2_ nanostructure enabling consecutive reversible charge transfer from Mn(IV) to Mn(II) in a mixed pseudocapacitor-battery electrode. Adv. Energy Mater. 5, 1500772 (2015).

[b42] Chen D. . Probing the charge storage mechanism of a pseudocapacitive MnO_2_ electrode using in operando Raman spectroscopy. Chem. Mater. 27, 6608–6619 (2015).

[b43] Ma R., Bando Y. & Sasaki T. Directly rolling nanosheets into nanotubes. J. Phys. Chem. B 108, 2115–2119 (2004).

[b44] Sun Z. . Generalized self-assembly of scalable two-dimensional transition metal oxide nanosheets. Nat. Commun. 5, 3813 (2014). 24814859 10.1038/ncomms4813

[b45] Omomo Y., Sasaki T., Wang L. & Watanabe M. Redoxable nanosheet crystallites of MnO_2_ derived via delamination of a layered manganese oxide. J. Am. Chem. Soc. 125, 3568–3575 (2003). 12643719 10.1021/ja021364p

[b46] Liu Z.-h., Ooi K., Kanoh H., Tang W.-p. & Tomida T. Swelling and delamination behaviors of birnessite-type manganese oxide by intercalation of tetraalkylammonium ions. Langmuir 16, 4154–4164 (2000).

[b47] Zhu M. . Structural study of biotic and abiotic poorly-crystalline manganese oxides using atomic pair distribution function analysis. Geochim. Cosmochim. Acta 81, 39–55 (2012).

[b48] Paek S.-M. . Exfoliation and reassembling route to mesoporous titania nanohybrids. Chem. Mater. 18, 1134–1140 (2006).

[b49] Song M.-S. . Porously assembled 2D nanosheets of alkali metal manganese oxides with highly reversible pseudocapacitance behaviors. J. Phys. Chem. C 114, 22134–22140 (2010).

[b50] Simon P., Gogotsi Y. & Dunn B. Where do batteries end and supercapacitors begin? Science 343, 1210–1211 (2014). 24626920 10.1126/science.1249625

[b51] Lew C. M. . Pure-silica-zeolite MFI and MEL low-dielectric-constant films with fluoro-organic functionalization. Adv. Funct. Mater. 18, 3454–3460 (2008).

[b52] Mao L., Zhang K., Chan H. S. O. & Wu J. Surfactant-stabilized graphene/polyaniline nanofiber composites for high performance supercapacitor electrode. J. Mater. Chem. 22, 80–85 (2012).

[b53] Kuo S.-L. & Wu N.-L. Investigation of pseudocapacitive charge-storage reaction of MnO_2_nH_2_O supercapacitors in aqueous electrolytes. J. Electrochem. Soc. 153, A1317–A1324 (2006).

[b54] Lee Y. R., Kim I. Y., Kim T. W., Lee J. M. & Hwang S. J. Mixed colloidal suspensions of reduced graphene oxide and layered metal oxide nanosheets: useful precursors for the porous nanocomposites and hybrid films of graphene/metal oxide. Chem. Eur. J. 18, 2263–2271 (2012). 22253000 10.1002/chem.201102646

[b55] Hwang S.-J. . Local crystal structure around manganese in new potassium-based nanocrystalline manganese oxyiodide. J. Phys. Chem. B 106, 4053–4060 (2002).

[b56] Egami T. & Billinge S. J. Underneath the Bragg Peaks: Structural Analysis of Complex Materials Elsevier (2003).

[b57] Metz P. . X-ray and neutron total scattering analysis of H_y_·(Bi_0.2_Ca_0.55_Sr_0.25_)(Ag_0.25_Na_0.75_)Nb_3_O_10_·xH_2_O perovskite nanosheet booklets with stacking disorder. Powder Diffr. 31, 126–134 (2016).

[b58] Wang H.-W., Naguib M., Page K., Wesolowski D. J. & Gogotsi Y. Resolving the structure of Ti_3_C_2_T_*x*_ MXenes through multilevel structural modeling of the atomic pair distribution function. Chem. Mater. 28, 349–359 (2015).

[b59] Oku M., Hirokawa K. & Ikeda S. X-ray photoelectron spectroscopy of manganese-oxygen systems. J. Electron. Spectrosc. Relat. Phenom. 7, 465–473 (1975).

[b60] Gao P. & Liu D. Facile synthesis of copper oxide nanostructures and their application in non-enzymatic hydrogen peroxide sensing. Sensors Actuators B: Chem. 208, 346–354 (2015).

[b61] Chang J. . Asymmetric supercapacitors based on graphene/MnO_2_ nanospheres and graphene/MoO_3_ nanosheets with high energy density. Adv. Funct. Mater. 23, 5074–5083 (2013).

[b62] Fan Z. . Asymmetric supercapacitors based on graphene/MnO_2_ and activated carbon nanofiber electrodes with high power and energy density. Adv. Funct. Mater. 21, 2366–2375 (2011).

[b63] Shao Y., Wang H., Zhang Q. & Li Y. High-performance flexible asymmetric supercapacitors based on 3D porous graphene/MnO_2_ nanorod and graphene/Ag hybrid thin-film electrodes. J. Mater. Chem. C 1, 1245–1251 (2013).

[b64] Huang M. . Self-assembly of mesoporous nanotubes assembled from interwoven ultrathin birnessite-type MnO_2_ nanosheets for asymmetric supercapacitors. Sci. Rep. 4, 3878 (2014). 24464344 10.1038/srep03878 PMC3902441

[b65] Wei W., Cui X., Chen W. & Ivey D. G. Manganese oxide-based materials as electrochemical supercapacitor electrodes. Chem. Soc. Rev. 40, 1697–1721 (2011). 21173973 10.1039/c0cs00127a

[b66] Ravel B. & Newville M. ATHENA, ARTEMIS, HEPHAESTUS: data analysis for X-ray absorption spectroscopy using IFEFFIT. J. Synchrotron Rad. 12, 537–541 (2005). 10.1107/S0909049505012719 15968136

[b67] Chupas P. J., Qiu X., Hanson J. C., Lee P. L. & Clare P. Rapid-acquisition pair distribution function (RA-PDF) analysis. J. Appl. Crystallogr. 36, 1342–1347 (2003).

[b68] Hammersley A., Svensson S., Hanfland M., Fitch A. & Hausermann D. Two-dimensional detector software: from real detector to idealised image or two-theta scan. High Pressure Res. 14, 235–248 (1996).

[b69] Qiu X., Thompson J. W. & Billinge S. J. PDFgetX2: a GUI-driven program to obtain the pair distribution function from X-ray powder diffraction data. J. Appl. Crystallogr. 37, 678–678 (2004).

[b70] Wojdyr M. Fityk : a general-purpose peak fitting program. J. Appl. Crystallogr. 43, 1126–1128 (2010).

