# Supplementary material for: The critical role of point defects in improving the specific capacitance of δ-MnO2 nanosheets
Source: Nat Commun. 2017 Feb 23;8:14559. doi: 10.1038/ncomms14559 (PMC5331340; doi:10.1038/ncomms14559)
Supplement: Supplementary Information — Supplementary Figures, Supplementary Table and Supplementary References [file ncomms14559-s1.pdf]

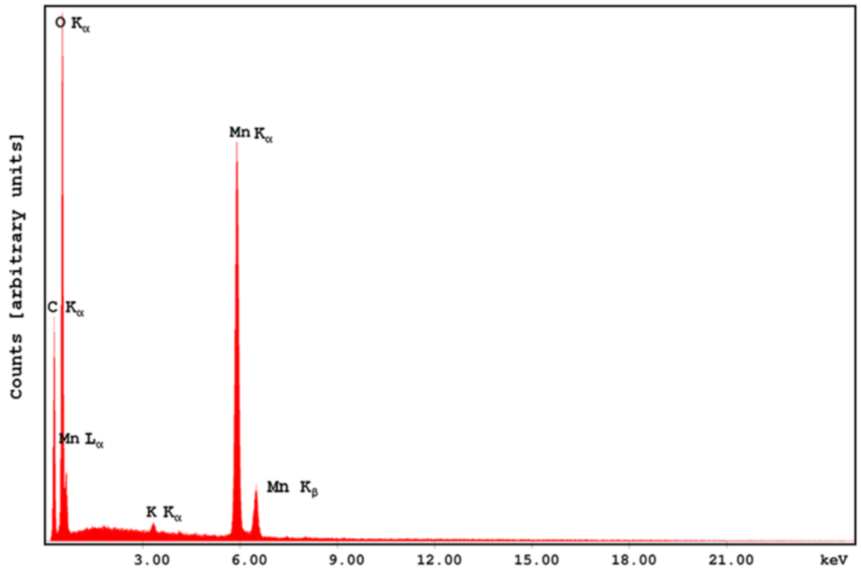

Supplementary Figure 1: Energy dispersive spectrum of protonated H_xMnO_2. The spectrum indicates negligible potassium quantities. Carbon signal is an artifact of the carbon tape used to prepare the powder mount.

Element	Wt%	At%
C	32.87	49.01
O	36.65	41.02
K	0.34	0.16
Mn	30.14	9.82

Supplementary Table 1: Standardless quantification of protonated H_xMnO_2. Quantification of the K from Supplementary Fig. 1 reveals substantially less than 1% residual K. Carbon signal is an artifact of the carbon tape used to prepare the powder mount.

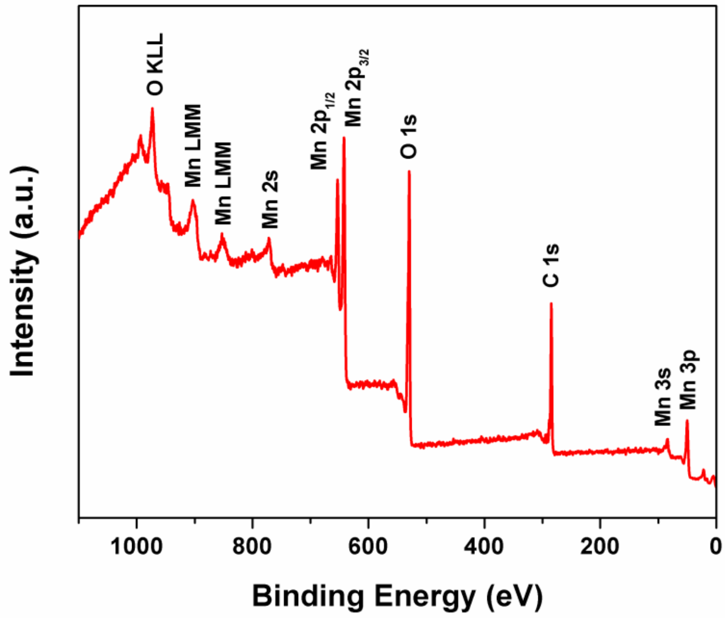

Supplementary Figure 2: XPS survey scan of protonated H_xMnO_2. The survey scan demonstrates the absence of potassium and aluminum peaks, which would be located at 292 and 76 eV, respectively.

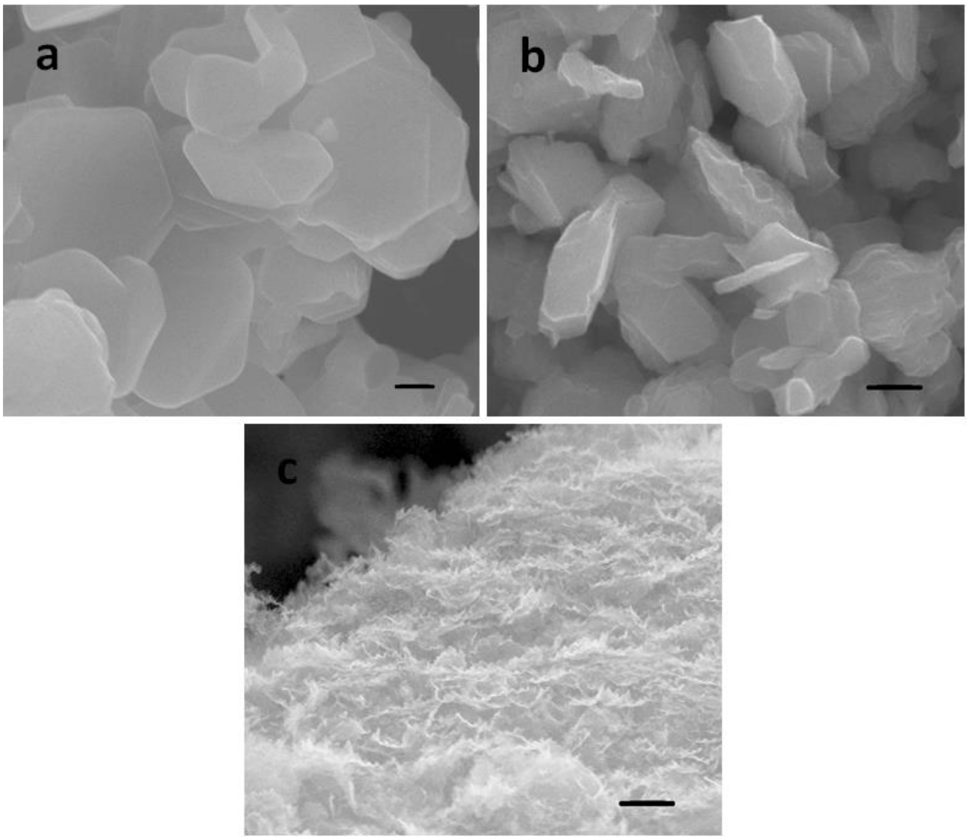

Supplementary Figure 3: Electron microscopy of nanosheet precursors and assembly. SEM images of (**a**) pristine K_xMnO_2, (**b**) protonated H_xMnO_2, and (**c**) reassembled MnO_2 treated in pH = 4 solution for 24 h. Scale bars, 500 nm (**a**-**c**).

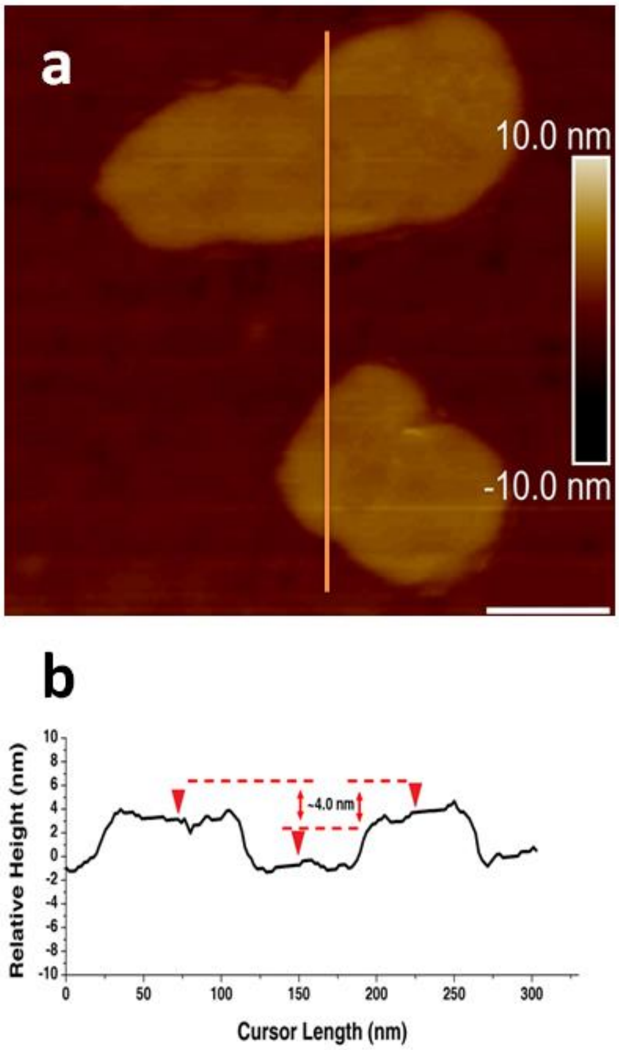

Supplementary Figure 4: Atomic force microscopy of MnO_2 nanosheets. (**a**) AFM image of MnO_2 nanosheets on Si wafer; (**b**) the height profile along the line shown in the AFM image in (**a**). Scale bar, 60 nm (**a**).

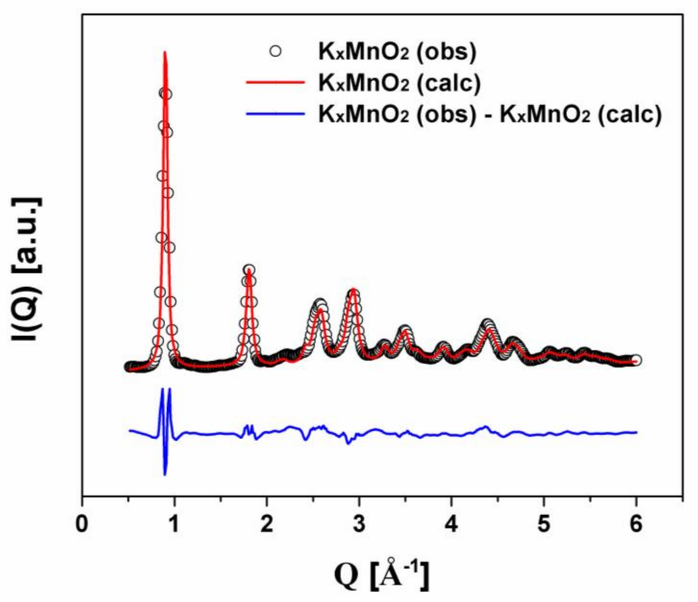

Supplementary Figure 5: Rietveld refinement of the parent compound K_xMnO_2. Rietveld refinement (R_{wp} = 13%) of the diffraction pattern of K_xMnO_2 synthesized by solid state methods reveals a mixture of monoclinic (C2/m, 91 %) and rhombohedral (R-3m, 9 %) birnessite polytypes. Data collected on APS 11-ID-B.

Phase purity of the starting compound was confirmed using synchrotron X-ray diffraction collected on APS 11-ID-B. While laboratory diffraction data tends to reveal little besides the *00l* reflections (Q < 2 $Å^{-1}$), high energy X-rays with high brilliance reveal more of the characteristic features hkl bands at larger scattering vectors. Scale factors for a monoclinic (C2/m) and a rhombohedral (R-3m) birnessite model were refined independently, while the lattice **c** parameter, isotropic thermal displacement, size, and strain variables were refined jointly for the two phases.

Little difference is observed in the resulting refinement, confirming the major crystalline phase is birnessite. The two birnessite polytypes differ only in regard to intersheet ordering, which is sensitive to the type and quantity of interlayer species present. The resulting protonated phase should therefore be insensitive to the polytypism observed.

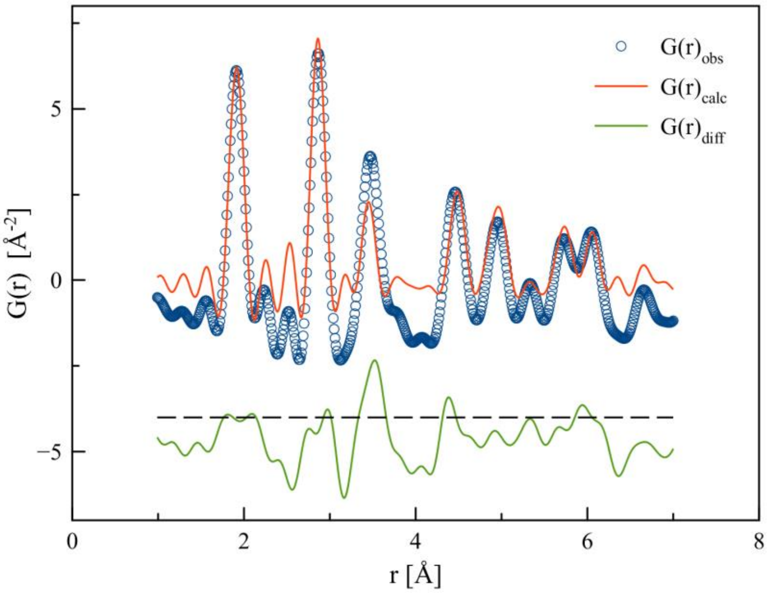

Supplementary Figure 6: Evidence for δ-phase MnO_2 nanosheets. Comparison of the experimental PDF (reassembled MnO_2, pH = 2) and the PDF calculated for a single δ-MnO_2 sheet confirms the δ-MnO_2 motif is maintained in the exfoliated and reassembled nanosheet floccs. The mismatched amplitude of the third major peak is a consequence of the Mn surface Frenkel defect, while the discrepancy in baseline is due to the comparison of an isolated sheet to a material with correlations in three dimensions.

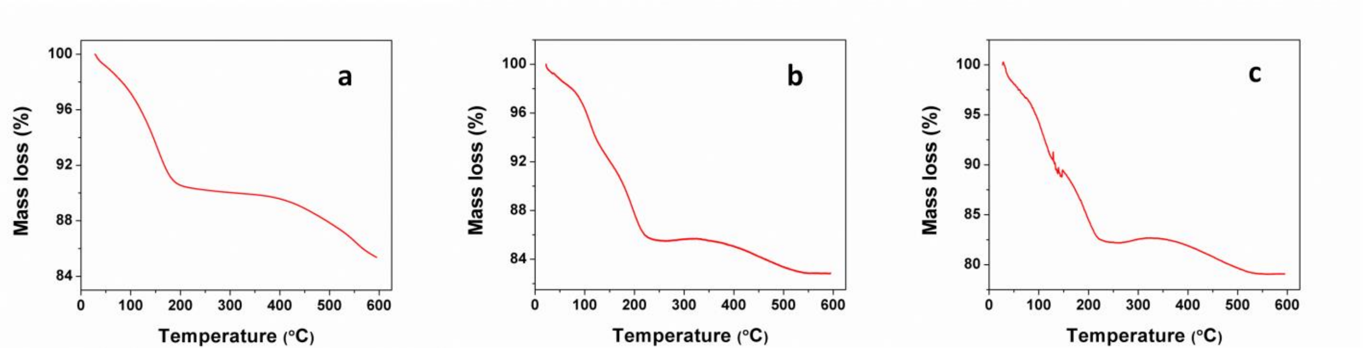

Supplementary Figure 7: Thermogravimetric analysis of H_xMnO_2 and nanosheet floccs. TGA curves of (**a**) protonated MnO_2, (**b**) reassembled MnO_2 treated in pH = 2 solution for 24 h, (**c**) reassembled MnO_2 treated in pH = 4 solution for 24 h.

The thermogravimetric analysis indicates 9.5 wt.% H_2O for H_xMnO_2 dried at 60°C (0.5 H_2O per MnO_2 formula unit), 12.3 wt.% (0.7 H_2O per MnO_2 formula unit) for the pH = 2 nanosheet assembly, and 15.5 wt.% (0.9 H_2O per MnO_2 formula unit) for the pH = 4 sample. An additional mass loss (~3 %) for the reassembled samples in the temperature region of 200-400 °C is assigned to decomposition of residual TBAOH, while at 400-600 °C all the samples show another mass loss corresponding to the evolution of oxygen from the lattice, leading to the formation of Mn_3O_4[1], and loss of structural hydroxyls[2].

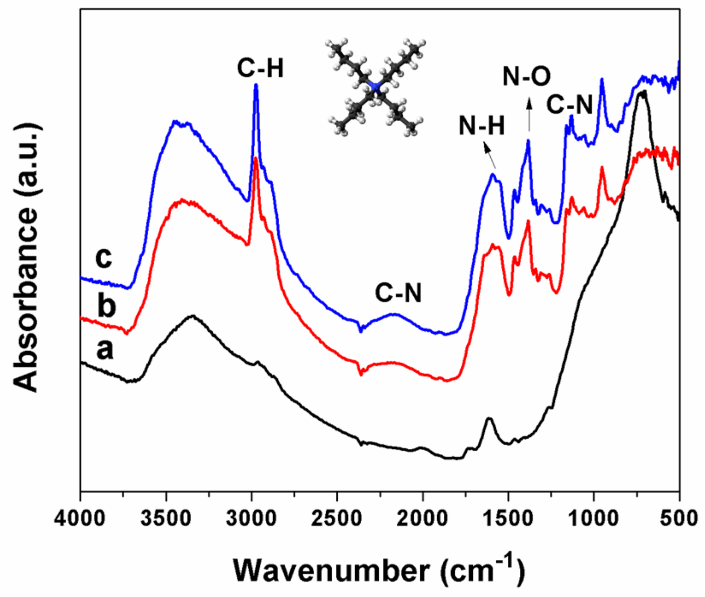

Supplementary Figure 8: FTIR spectra of H_xMnO_2 and nanosheet assemblies. FTIR spectra of (**a**) protonated MnO_2; (**b**) reassembled MnO_2 treated in pH = 4 solution for 24 h; (**c**) reassembled MnO_2 treated in pH = 2 solution for 24 h (inset shows the structure of the TBAOH molecule).

As shown in Supplementary Fig. 8, all the sharp bands near 2980 cm^{-1} belong to C-H stretching vibrations in TBAOH molecules. Direct observation of nitrogen via the C-N vibration is apparent in a broad IR band near 2000 - 2300 cm^{-1} while the bands in the 950 - 1300 cm^{-1} region correspond to the C-N stretching vibration. The bands located at 1560 and 1600 cm^{-1} are a result of the N-H stretching vibration, and that at 1385 cm^{-1} is related to the N-O stretching vibration [3, 4, 5, 6].

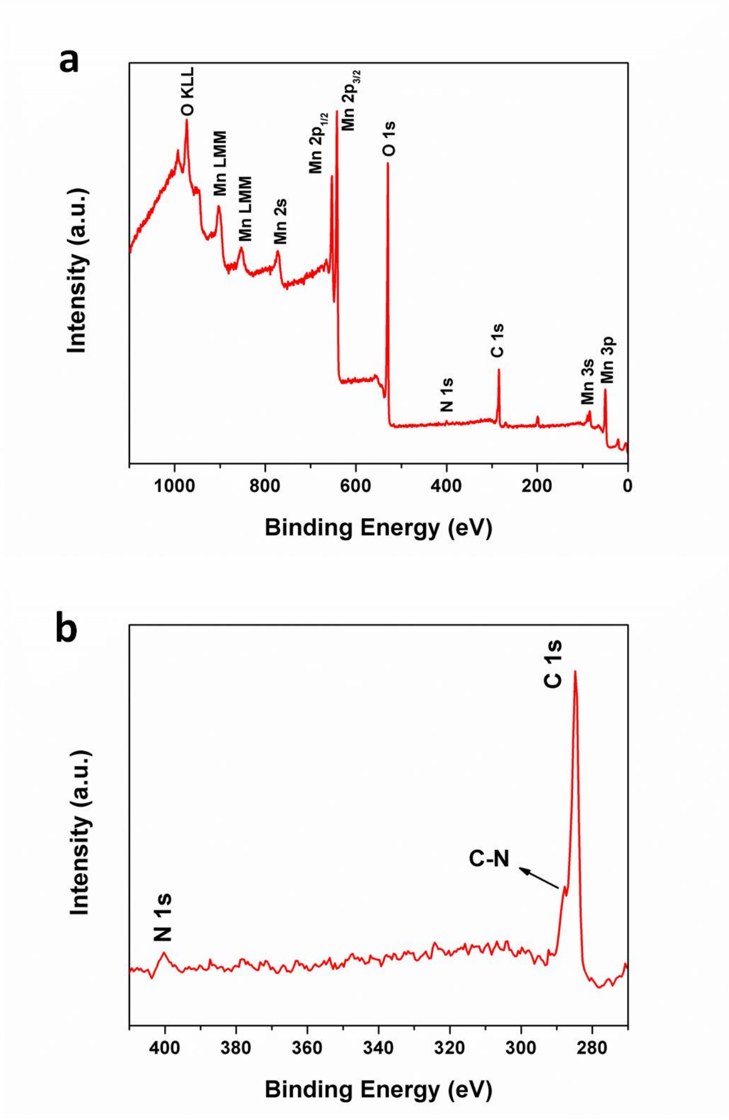

Supplementary Figure 9: Detection of TBAOH using XPS. XPS spectra of reassembled MnO_2 treated in pH = 2 solution for 24 h. (**a**) XPS survey scan, (**b**) high resolution XPS N 1s and C 1s spectrum.

The presence of residual TBAOH in the reassembled samples has been confirmed by using the XPS survey scan, which we can clearly see the N 1s peak coming from the TBAOH molecules. Besides, the non-symmetrical C 1s peak shape shown in Supplementary Fig. 9(**b**), as well as the presence of the small peak located at around 288 eV that can be attributed to carbon bounded to nitrogen[7], also confirmed the existence of TBAOH after exfoliation and flocculation processes.

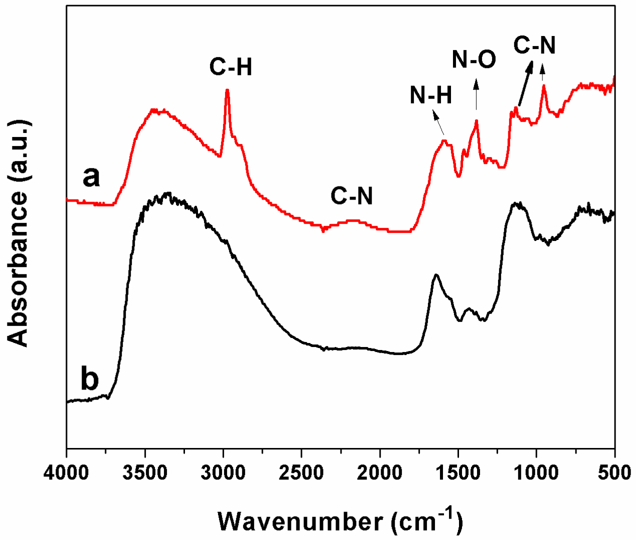

Supplementary Figure 10: FTIR spectra of reassembled δ-MnO_2. FTIR spectra of reassembled MnO_2 treated in pH = 4 solution for 24 h, (**a**) before and (**b**) after one CV cycling in 1M Na_2SO_4 electrolyte at 50 mV·s^{-1} scan rate.

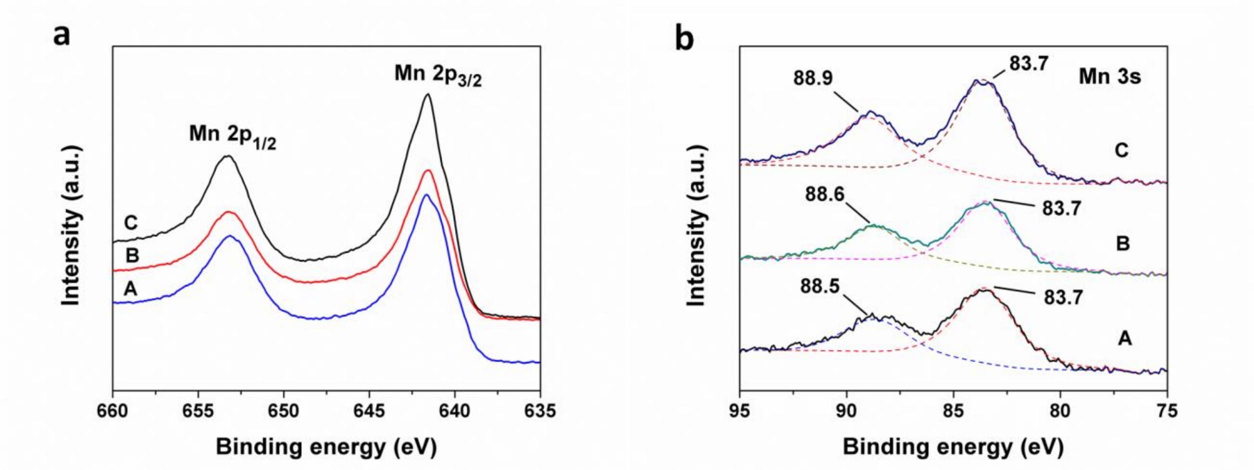

Supplementary Figure 11: High-resolution XPS of the Mn 2p and 3s regions. XPS spectra in the (**a**) Mn 2p, and (**b**) Mn 3s regions corresponding to (**A**) protonated MnO_2, **(B)** reassembled MnO_2 treated in pH = 4 solution for 24 h, (**C**) reassembled MnO_2 treated in pH = 2 solution for 24 h.

The chemical environment/oxidation state of Mn was analyzed using X-ray photoelectron spectroscopy (XPS). As shown in Supplementary Figure 11(**a**), the Mn 2p region mainly consists of a spin-orbit doublet corresponding to the Mn $2p_{1/2}$ and Mn $2p_{3/2}$ states around the binding energy of 653.0 and 642.0 eV. Generally speaking, the binding energy values can be used to roughly estimate the oxidation state of manganese. According to the literature, binding energy values of 642.0 eV are usually assigned to Mn^{4+}, 641.0 eV to Mn^{3+}, and 640.0 eV to Mn^{2+} [8, 9]. Since the main Mn $2p_{3/2}$ peak for all samples is located between 642.0 and 641.0 eV, and the shape of the Mn $2p_{3/2}$ peaks is obviously not symmetrical, which implies the coexistence of Mn^{3+} and Mn^{4+} in the MnO_2 nanosheets. Moreover, studies of Biesinger *et al.*[10] showed that multiple peaks resolved under the Mn 2p spectrum can represent a single oxidation state. Thus, due to the complexity of oxidation states in Mn 2p spectrum as well as the lack of standardization, it is difficult to determine the manganese oxidation state only from the Mn $2p_{3/2}$ peak. The splitting of Mn 3s peaks is often used to determine the oxidation state of Mn[11, 12, 13], where the electron exchange interaction

upon photoelectron ejection defines the magnitude of the splitting. The separation of peak energies for the electron exchange in the 3s-3d level of Mn is described by Eq. S1.

$$\Delta E = (2S + 1)\, K\, [3s, 3d] \qquad \text{S1}$$

where ΔE is the separation of peak energies. S is the total spin of unpaired electrons in the 3s and 3d levels in the final states and K [3s, 3d] is the exchange integral between 3s-3d energy levels. Based on the above theory, lower valence of Mn will lead to wider splitting of the 3s peaks. Therefore, we are able to qualitatively compare the Mn^{3+} content in the three samples through study the Mn 3s peak energy separation as shown in Supplementary Figure 11(**b**). The ΔE values obtained are 4.8 eV for the protonated MnO_2, 4.9 eV for the pH = 4 treated reassembled MnO_2, and 5.2 eV for the pH = 2 treated reassembled MnO_2. The results indicate that lower pH treatment of the reassembled MnO_2 nanostructures can lead to the formation of more Mn^{3+} in the MnO_2 nanosheet. The protonated MnO_2 exhibits a lower Mn^{3+} content compared with both the pH = 2 and 4 samples, despite being treated at lower pH. This apparent contradiction results from steric effects in the crystalline H_xMnO_2 that inhibit Mn occupation of the interlayer galleries (see main text for detailed discussion).

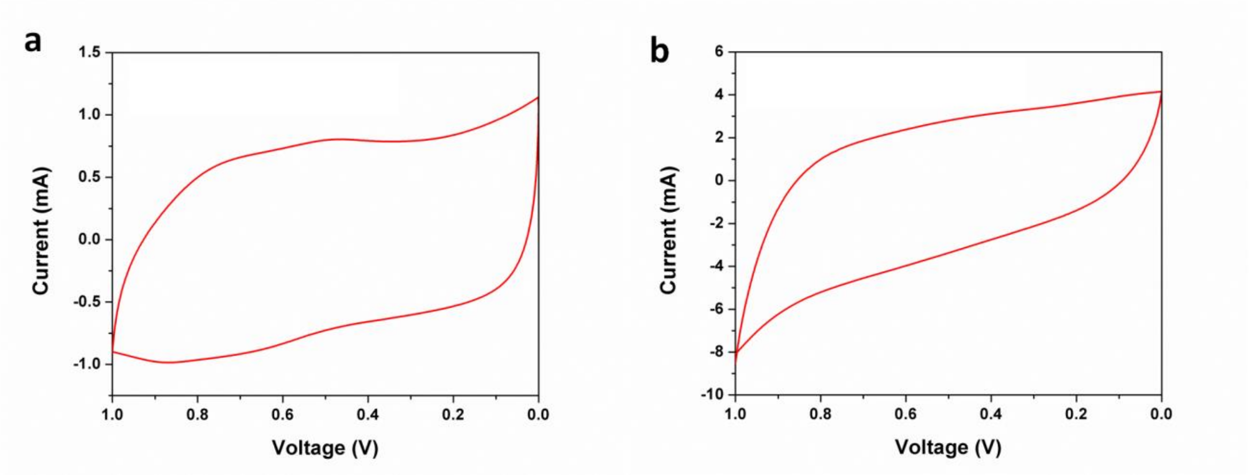

Supplementary Figure 12: Cyclic voltammetry of reassembled δ-MnO_2 equilibrated at pH 3 and 9.

Cyclic voltammetry curves for nanosheet assemblies treated at (**a**) pH = 3 and (**b**) pH = 9.

Supplementary References:

1. Lee, Y.R., Kim, I.Y., Kim, T.W., Lee, J.M., Hwang, S.J. Mixed colloidal suspensions of reduced graphene oxide and layered metal oxide nanosheets: useful precursors for the porous nanocomposites and hybrid films of graphene/metal oxide. *Chem. Eur. J.* **18**, 2263-2271 (2012).

2. Wang, J., Zhang, P., Li, J., Jiang, C., Yunus, R., Kim, J. Room-temperature oxidation of formaldehyde by layered manganese oxide: effect of water. *Environ. Sci. Technol.* **49**, 12372-12379 (2015).

3. Burguete, M.I., Gavara, R., Galindo, F., Luis, S.V. New polymer-supported photocatalyst with improved compatibility with polar solvents. Synthetic application using solar light as energy source. *Catal. Commun.* **11**, 1081-1084 (2010).

4. Argazzi, R., Larramona, G., Contado, C., Bignozzi, C.A. Preparation and photoelectrochemical characterization of a red sensitive osmium complex containing 4, 4′, 4′′-tricarboxy-2, 2′ : 6′, 2′′-terpyridine and cyanide ligands. *J. Photochem. Photobiol. A: Chem.* **164**, 15-21 (2004).

5. Medvecký, Ľ., Briančin, J., Ďurišin, J. Nanohydroxyapatite prepared by rapid precipitation method in the presence of tetrabutylammonium hydroxide. *Powder Metall. Prog.* **10**, 213 (2010).

6. Andrade, Â.L., Fabris, J.D., Ardisson, J.D., Valente, M.A., Ferreira, J.M. Effect of tetramethylammonium hydroxide on nucleation, surface modification and growth of magnetic nanoparticles. *J. Nanomater.* **2012**, 15 (2012).

7. Matsuoka, M., *et al.* X-ray photoelectron spectroscopy and raman spectroscopy studies on thin carbon nitride films deposited by reactive RF magnetron sputtering. *World Journal of Nano Science and Engineering* **2**, 92 (2012).

8. Nakayama, M., Tanaka, A., Konishi, S., Ogura, K. Effects of heat-treatment on the spectroscopic and electrochemical properties of a mixed manganese/vanadium oxide film prepared by electrodeposition. *J. Mater. Res.* **19**, 1509-1515 (2004).

9. Nakayama, M., Konishi, S., Tagashira, H., Ogura, K. Electrochemical synthesis of layered manganese oxides intercalated with tetraalkylammonium ions. *Langmuir* **21**, 354-359 (2005).

10. Biesinger, M.C., Payne, B.P., Grosvenor, A.P., Lau, L.W., Gerson, A.R., Smart, R.S.C. Resolving surface chemical states in XPS analysis of first row transition metals, oxides and hydroxides: Cr, Mn, Fe, Co and Ni. *Appl. Surf. Sci.* **257**, 2717-2730 (2011).

11. Chigane, M., Ishikawa, M. Manganese oxide thin film preparation by potentiostatic electrolyses and electrochromism. *J. Electrochem. Soc.* **147**, 2246-2251 (2000).

12. Carver, J., Schweitzer, G., Carlson, T.A. Use of X‐ray photoelectron spectroscopy to study bonding in Cr, Mn, Fe, and Co compounds. *J. Chem. Phys* **57**, 973-982 (1972).

13. Oku, M., Hirokawa, K., Ikeda, S. X-ray photoelectron spectroscopy of manganese-oxygen systems. *J. Electron. Spectrosc. Relat. Phenom.* **7**, 465-473 (1975).
